# In-Situ Conversion of ZnO/Ni_3_ZnC_0.7_/CNT Composite from NiZn Bimetallic MOF Precursor with Enhanced Electromagnetic Property

**DOI:** 10.3390/nano8080600

**Published:** 2018-08-07

**Authors:** Lina Huang, Shaolong Huang, Ziyu Yang, Ailun Zhao, Chengxiang Liu, Jianguo Lu, Shuangchen Ruan, Yu-jia Zeng

**Affiliations:** 1 Shenzhen Key Laboratory of Laser Engineering, Guangdong Provincial Key Laboratory of Micro/Nano Optomechatronics Engineering, College of Optoelectronic Engineering, Shenzhen University, Shenzhen 518060, China; huanglina007@sina.com (L.H.); nkhsl3313@163.com (S.H.); yangziyu@pku.edu.cn (Z.Y.); ailunzhao@outlook.com (A.Z.); scruan@szu.edu.cn (S.R.); 2 College of Mechatronics and Control Engineering, Shenzhen University, Shenzhen 518060, China; chxliu@szu.edu.cn; 3 State Key Laboratory of Silicon Materials, School of Materials Science and Engineering, Zhejiang University, Hangzhou 310027, China; lujianguo@zju.edu.cn

**Keywords:** MOF, electromagnetic absorbing properties, CNT, ZnO

## Abstract

ZnO/Ni_3_ZnC_0.7_/x% CNT (x = 0, 2, 5, 10) urchin-like structures have been synthesized using a facile method based on metal-organic frameworks (MOFs) and applied as the electromagnetic absorber. The results of the experiments demonstrate that the content of multi-wall carbon nanotubes (MWCNT) has a great influence on the absorbing performance of the hybrid material. Conduction loss, interfacial polarization and ferromagnetic resonance all contribute to the electromagnetic absorption. The urchin-like ZnO/Ni_3_ZnC_0.7_/5% CNT composite presents excellent absorbing properties. When the filler loading of ZnO/Ni_3_ZnC_0.7_/5% CNT composite in paraffin matrix is only 10 wt.%, a minimum reflection loss of −33.2 dB is achieved at a layer thickness of 4.9 mm.

## 1. Introduction

Lightweight microwave absorbing materials with the ability of converting the electromagnetic (EM) energy to the thermal energy have wide applications in both environment and military [1,2,3]. It becomes widely known that the traditional microwave absorbers usually suffer from issues including narrow absorption bandwidth, high filler loading, and large thickness, which largely restrain their engineering applications [4,5,6,7,8]. Hence, rational design and fabrication of novel absorbers with excellent comprehensive performance have stimulated extensive attention recently. 

In recent years, many advanced approaches have been adopted for improving microwave absorbing properties, such as improving impedance matching by synthesizing magnetic-dielectric nanocomposite, decreasing weight density by fabricating porous/hollow structured materials, and enhancing interfacial area by constructing hetero-interfaces. Metal-organic frameworks (MOFs) have been considered as an ideal precursor/template for magnetic nanoparticles/carbon composite absorbers since the report on the synthesis of Fe/C composite absorber from Prussian blue [9]. MOFs possess desirable advantages, i.e., porous structures, abundant hetero-interfaces, magnetic and dielectric dual losses, and large specific area. Until now, multiple MOF-derived composite absorbers such as ZnO/Fe/Fe_3_C/C [10], Co_3_O_4_/Co/RGO [11], ZnO/SiC [12], ZnO/Fe [13], ZnO/Co_3_ZnC/Co/C [14] have been fabricated and have been shown to exhibit good microwave absorbing properties. However, the filler loading in matrix of these recent MOF-derived absorbers is generally more than 40 wt.%, which fails to fulfill the lightweight requirement for new-generation electromagnetic absorbers. To overcome the above problems, researchers have initiated some strategies, i.e., mixing two or more components to achieve the synergistic effect by the reinforcement of every component. 

Further, good impedance matching which derives from the complementarity between permittivity and permeability is important for excellent microwave absorption [15]. Therefore, controlling the ratio between magnetic and dielectric components is critical for the wave-absorbing performance. Nevertheless, the content of magnetic component in previous MOF derived absorbers is quite limited because of the fixed MOF composition. To match the permittivity and permeability, a modified method to fabricate composites with multi-wall carbon nanotubes (MWCNT) has been reported through a multiple-step synthesis: fabricating mixed free MOF, mixing MWCNT with MOF particles, and post annealing. Metal oxides are believed to result in the decrease of the electrical conductivity for improving the impedance matching [16,17,18,19]. As well, metal carbides can be introduced to the composites which result in an outstanding magnetic absorbing property. 

In this paper, a feasible and facile strategy has been demonstrated to synthesize ZnO/Ni_3_ZnC_0.7_/CNT composite from MOF precursors. Being different from previous materials, the hybrids have extremely low filler loading, which is only 10 wt.%. The metallic Ni is responsible for the magnetic loss in the composite, ZnO phase contributes to the conducive loss, and CNT phase contributes to the dielectric loss. Different phases are all favorable for electromagnetic waves attenuation [20]. By optimizing the composition and microstructure of the ZnO/Ni_3_ZnC_0.7_/CNT composite derived from bimetallic MOF, the composite with a filler loading of only 10 wt.% exhibits a strong microwave absorption with a minimum reflection loss (RL) of −33.2 dB. 

## 2. Experimental Section

Nickle nitrate hexahydrate (Ni(NO_3_)_2_·6H_2_O, 99%), zinc nitrate hexahydrate (Zn(NO_3_)_2_·6H_2_O, 99%), 2,5-dihydroxyterephthalic acid (98%), ethylene glycol (EG) (99%), *N*,*N*-dimethylformamide (DMF), and ethanol were purchased from Shanghai Aladdin. All reagents were used without further purification with the analytical class.

### 2.1. Pre-Treatment of Carbon Nanotubes

The MWCNTs used in this work were purchased from Beijing Zhongxingbairui Technoloy Co., Ltd. (Beijing, China), with the length of several micrometers and the diameter of about 15 nm. First, 3.0 g of CNT was refluxed in 90 mL of concentrated HNO_3_ at 120 °C for 6.5 h. Second, the treated CNTs were rinsed with DI water for several times until achieving a neutral PH value. Finally, the content was gathered by filtration and centrifugation and dried at 50 °C for next use.

### 2.2. Synthesis of Urchin-Like Ni-Zn MOF /x% CNT (x = 0, 2, 5, 10) Composite

First, 0.005 g (2 wt.%), 0.0125 g (5 wt.%), and 0.025 g (10 wt.%) CNT were dissolved in 5 mL DMF and stirred for 1 h at room temperature. Second, 0.3 g of 2,5-dihydroxyterephthalic acid and 0.5 g of Zn(NO_3_)_2_·6H_2_O and Zn(NO_3_)_2_·6H_2_O were dissolved in a mixed solvent of 80 mL of *N*,*N*-dimethylformamide (DMF), and 50 mL of ethylene glycol, then stirred the mixing solution for about 1 h at room temperature. The processed CNT was added to the solution derived from step 2, then stirred for 1 h at room temperature. After that, the mixture was transferred to an autoclave made of teflon-lined stainless steel and was maintained at 150 °C for 8 h. The precipitation was washed with DMF firstly and then with ethanol for several times after cooling to room temperature. Lastly, the substance was dried in vacuum with the temperature of 60 °C all the night for use.

### 2.3. Synthesis of ZnO/Ni_3_ZnC_0.7_/x% CNT (x = 0, 2, 5, 10) Composites

The obtained urchin-like Ni-Zn MOF/x% CNT (x = 0, 2, 5, 10) composites were annealed in an H_2_/Ar atmosphere at 550 °C for 2 h at a heating rate of 5 °C/minute. Then the ashen powders were collected at room temperature. The final ZnO/Ni_3_ZnC_0.7_/x% CNT (x = 0, 2, 5, 10) composites were denoted as 0% CNT, 2% CNT, 5% CNT, and 10% CNT respectively, according to the CNT weight percentage of 0 wt.%, 2 wt.%, 5 wt.%, and 10 wt.%.

### 2.4. Characterizations

The crystal structure of the samples was analyzed by X-ray diffractometer (XRD, D8 Advance) with Cu Kα irradiation. Scanning electron microscopy (SEM, JEOL, JSM 7500, Tokyo, Japan) equipped with energy-dispersive X-ray spectroscopy and transmission electron microscopy (TEM, JEOL, JEM-2100, Tokyo, Japan) were used for characterizing the microstructure, morphology, as well the composition ratio of the composites. Raman spectra (Renishaw, INVIA REFLEX, Wotton-under-Edge, England, UK) were measured by using a 532 nm laser. Elemental valence state and composition of the samples were confirmed by using X-ray photoelectron spectrometer (VG, ESCALab 220i-XL, Milford, CT, USA). N_2_ adsorption-desorption was measured by Brunauer-Emmett-Teller test with a physisorpion analyzer (Micromeritics, ASAP2460, Norcross, GA, USA). The static magnetic properties of the composites were measured by using a vibrating sample magnetometer at room temperature.

### 2.5. Measurement of Electromagnetic Property

The electromagnetic parameters: relative complex permittivity and permeability of the ZnO/Ni_3_ZnC_0.7_/x% CNT (x = 0, 2, 5, 10) samples were tested using an Agilent E5062A network analyzer in the frequency range of 2–18 GHz. To prepare testing samples, the ZnO/Ni_3_ZnC_0.7_/x% CNT (x = 0, 2, 5, 10) composites powders were uniformly dispersed in paraffin matrix with a weight percentage of just 10 wt.%. Then the mixtures were pressed into toroidal shape with an outer diameter of 7.00 mm, an inner diameter of 3.04 mm and a thickness of 2.0 mm.

## 3. Results and Discussion

### 3.1. Structural Characterization and Magnetic Properties

**Figure 1:**
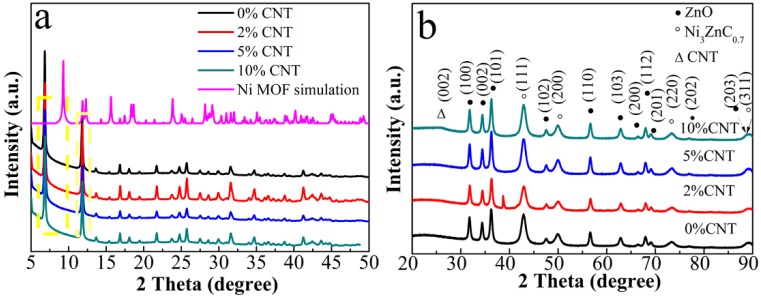
X-ray diffraction (XRD) patterns of (**a**) precursor of NiZn MOF; (**b**) ZnO/Ni3ZnC0.7/x% CNT (x = 0, 2, 5, 10) composites.

**Figure 2:**
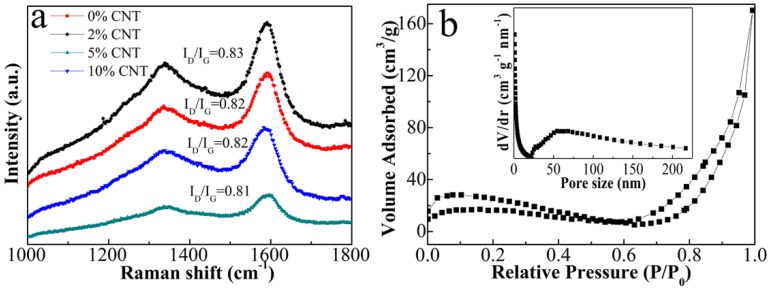
(**a**) Raman spectra of the composites with different contents of CNTs; (**b**) nitrogen adsorption/desorption isotherm of 5% CNT.

Figure 1a indicates the XRD modes of the Zn-doped MOF precursors with different contents of CNT. All samples present similar patterns, which suggests that all precursor MOFs have a layered structure of [Ni_3_(OH)_2_(C_8_H_4_O_4_)_2_(H_2_O)_4_]·2H_2_O. However, the diffraction angles 2θ shifts toward a lower angle, which might be due to the fact that the small Ni^2+^ ions are replaced or half part replaced by the large Zn^2+^ ions and is consistent with the previous substituted MOF [21]. As displayed in Figure 2b, XRD patterns of as-prepared composites demonstrate that the product is composed of ZnO (JCPDF no. 89-0510), Ni_3_ZnC_0.7_ (JCPDF no. 28-0713) [18] and CNT (26.2°). No impurity related peak is detected, suggesting the high pureness of the composites. 

In Figure 2a, we present Raman spectra of ZnO/Ni_3_ZnC_0.7_/x% CNT (x = 0, 2, 5, 10) composites. Two representative peaks at about 1345 cm^−1^ and 1580 cm^−1^ are commonly associated with disordered carbon or finite-size graphitic structure and the graphitic layers, respectively [22]. In general, the intensity ratio of D band to G band (*I*_D_/*I*_G_) is used to evaluate the graphitization degree of carbon materials. As the CNT content increases to 10%, the value of *I*_D_/*I*_G_ is almost constant, which demonstrates that the content of CNT will not affect the graphitization of carbon component during the pyrolysis process. The well-graphitized carbon is favorable to the electrical conductivity as well as the absorption of electromagnetic wave.

The nitrogen adsorption isotherm with an obvious hysteresis loop is a typical IV-type curve in the range of ca. 0.8–1.0 P/P_0_ (Figure 2b) [23]. The BET surface area of 5% CNT composite is measured to be 80.96 cm^2^/g. In addition, the inset of Figure 2b shows the Barrett-Joyner-Halenda (BJH) pore size distribution curve that displays the distribution of the pore size in 5% CNT.

**Figure 3:**
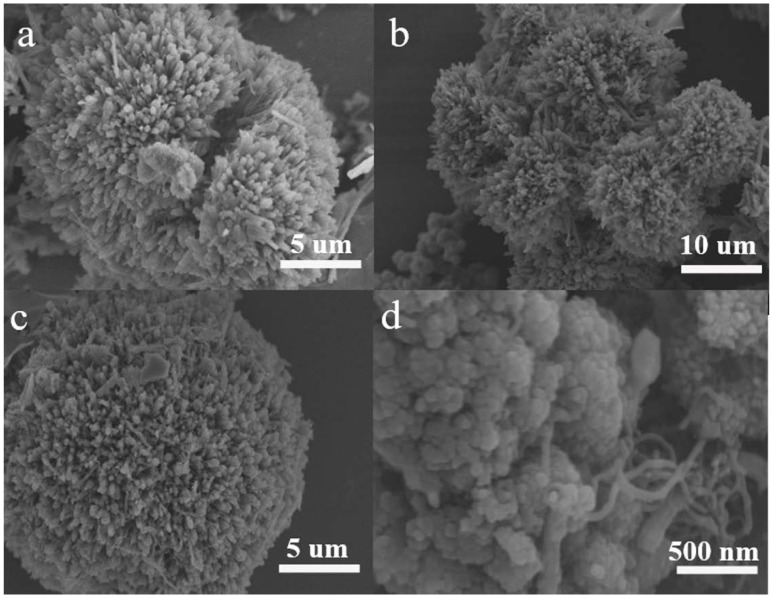
Scanning electron microscopy (SEM) images of (**a**) NiZn metal-organic framework (MOF) precursor without CNT; (**b**,**c**) 0% CNT; (**d**) 5% CNT.

**Figure 4:**
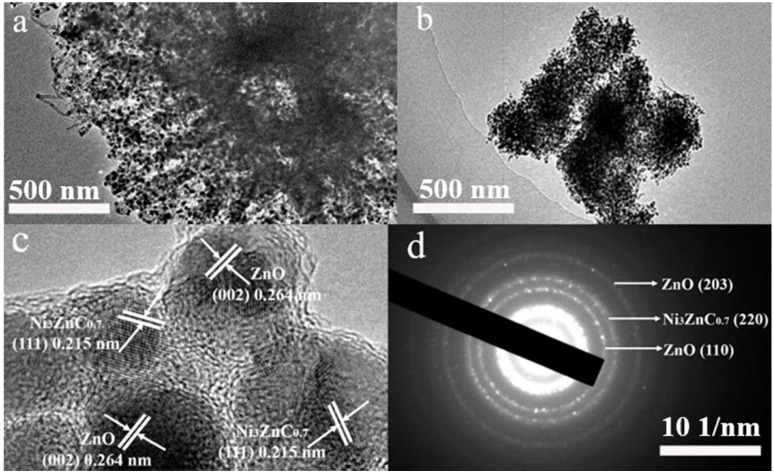
Transmission electron microscopy (TEM) graphics of (**a**) 0% CNT; (**b**) 5% CNT composite and (**c**) HR-TEM images of Ni_3_ZnC_0.7_ and ZnO particles in 5% CNT composite; (**d**) the corresponding SAED patterns.

The morphology of the precursor MOF and as-prepared 0% CNT and 5% CNT composites are imaged by both SEM and TEM. As demonstrated in Figure 3a, the MOF precursor shows an urchin-like structure and its particle size is about 10 μm. After pyrolysis, the morphology retains almost the same as shown in Figure 3b–d. The tangle-like CNT can effectively enhance the conductivity of 5% CNT composite. Additionally, there are plentiful of ZnO and Ni_3_ZnC_0.7_ nano-particles (NPs) diffusing in the composites as shown in Figure 4a,b. The marked lattice spacing of 0.215 nm and 0.52 nm in the 5% CNT composite with a high-resolution TEM lattice image matches well with the plane spacing of Ni_3_ZnC_0.7_ (111) and ZnO (001), respectively (Figure 4c) [24,25]. In addition, some spot-connected rings or diffraction spots of ZnO and Ni_3_ZnC_0.7_ can be seen in Figure 4d, which can be indexed to the (203) and (110) planes of ZnO and the (220) plane of Ni_3_ZnC_0.7_. All these results demonstrate that the urchin-like ZnO/Ni_3_ZnC_0.7_/CNT composites have been synthesized by the carbonization of the NiZn precursor MOF. 

Figure S1a–d show O, C, Ni and Zn EDX elemental mappings, which demonstrate Ni, Zn, O and C elements are evenly distributed in the ZnO/Ni_3_ZnC_0.7_@CNT composite. From these results, it can be further confirmed that the hybrid materials are synthesized successfully. In addition, EDS characterizations are performed on the four samples to reveal their compositions as shown in Table S1. 

**Figure 5:**
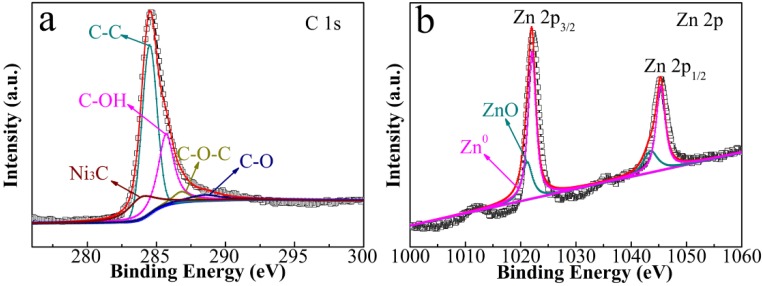
XPS spectra of 5% CNT composite: the deconvolution of (**a**) C1s; (**b**) Zn2p spectra.

In Figure 5a, we show XPS results of the representative 5% CNT composite. The survey spectrum is shown in Figure S2a. The deconvolution of C1*s* spectrum reveals five components that coincide with different carbon-containing functional groups: C-Ni (283.9 eV), C–C (284.5 eV), C–OH (285.7 eV), C–O–C (286.8 eV), and C–O (288.5 eV). Figure 5b provides Zn2*p* XPS spectrum, which contains primary peaks of Zn 2*p*_3/2_ and Zn 2*p*_1/2_ [26,27]. The deconvolution result reveals the coexistence of two Zn species, i.e., Zn-Ni with binding energies at 1021.17 and 1043.5 eV as well as ZnO at 1022.05 and 1045.5 eV. The crystal structure of Ni_3_ZnC_0.7_ is shown in Figure S2b, which can further illustrate the valence of Zn element in the composite due to the existing Ni-Zn bonds in the Ni_3_ZnC_0.7_.

**Figure 6:**
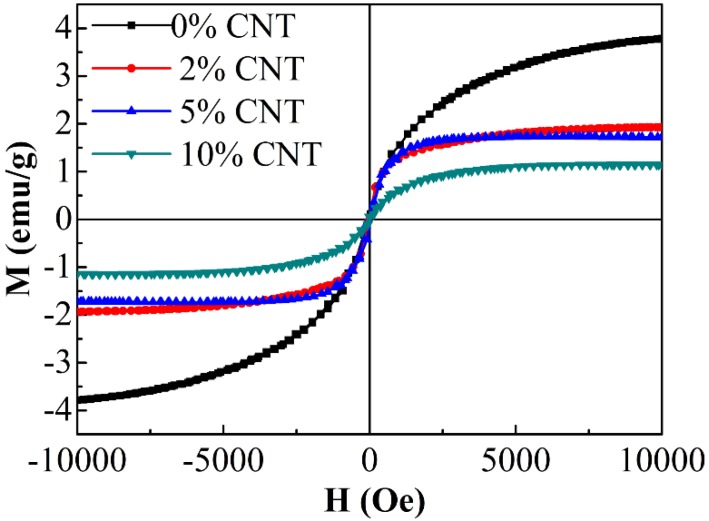
Field-dependent magnetizations of the composites.

The magnetization curves of the composites are measured by VSM as presented in Figure 6. After adding CNT, the saturation magnetization of the all samples decreases. The small values of the saturation magnetization and coercivity (*H_c_*) demonstrate their ferromagnetic characteristics. The increment of CNT (5%, 10%) content in composites intensively decreased the magnetic moment and caused the reduction of *M_s_* from 3.79 to 1.15 emu/g whereas the *H_c_* values are almost constant.

### 3.2. EMW Absorption Properties and Mechanism Analysis

**Figure 7:**
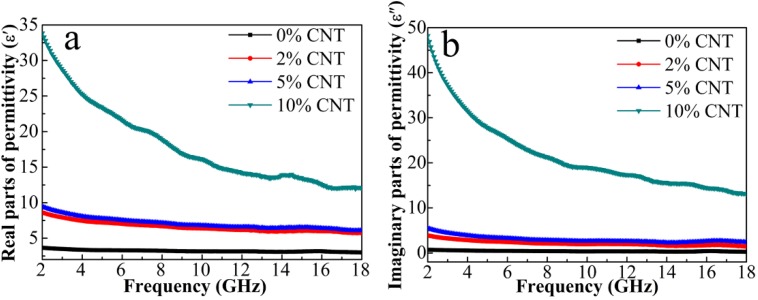
(**a**) Real parts and (**b**) imaginary parts of permittivities for the composites.

The relative complex permittivities and relative complex permeabilities of the composites have been measured in 2–18 GHz to evaluate the microwave absorbing properties. Figure 7a shows that the *ε*′ curve of the 0% CNT composite is almost a straight line, which does not vary with the frequency. On the other hand, after complexing CNT, all curves show a decline trend with increasing frequency due to the increased lagging of polarization with respect to the change of electric-field at higher frequency range [28]. The 10% CNT sample has the biggest drop with the increase of the frequency.

As the content of CNT increases, the *ε′* value also increases gradually. Generally, a medium *ε′* value in the range of 8–20 is beneficial for excellent impedance matching whereas a small *ε′* value below 6 makes EM wave easily penetrate rather than being attenuated [29]. On the basis of the microwave absorption mechanism, the imaginary part of permittivity represents dielectric loss, where a larger *ε″* value maintains a stronger microwave attenuation. It is evident from Figure 7b that the *ε″* value increases with the CNT content, with a maximum value for 10% CNT. The enhanced *ε″* value is associated with the increased polarization and electrical conductivity according to the equation $\varepsilon \prime \prime =\frac{\varepsilon_{s}-\varepsilon_{\infty }}{1+\omega^{2}\tau^{2}}\omega \tau +\frac{\sigma }{\omega \varepsilon_{0}}$ where *ε_s_*, *ε_∞_*, *ε*_0_ is the static permittivity, the relative permittivity at the high-frequency limit, and the permittivity of free space, respectively. Among them, *σ*, *τ*, and *ω* are the electrical conductivity, relaxation time, and angular frequency, respectively [30,31]. Specifically, the content of semi-conductive ZnO and the increased content of more conductive CNT are all contribute to the electrical conductivity. The *ε″* values are mainly enhanced by the *σ* value of the 3D network and the polarization between the interfaces, such as ZnO/Ni_3_ZnC_0.7_-CNT, CNT-carbon, carbon-ZnO/N_i3_ZnC_0.7_, as well as the defect-dipoles generated by the oxygen vacancies in ZnO nanoparticles (NPs).

Generally, interfacial polarization and dipole polarization dominate the permittivity in gigahertz frequency. Hence, we analyzed the polarization relaxation processes based on Debye relaxation theory, and *ε′* and *ε″* should obey the formula [32,33]

(1) $${\left(\varepsilon^{\prime }-\varepsilon_{\infty }\right)}^{2}+{\left(\varepsilon \prime \prime \right)}^{2}={\left(\varepsilon_{s}-\varepsilon_{\infty }\right)}^{2}$$

**Figure 8:**
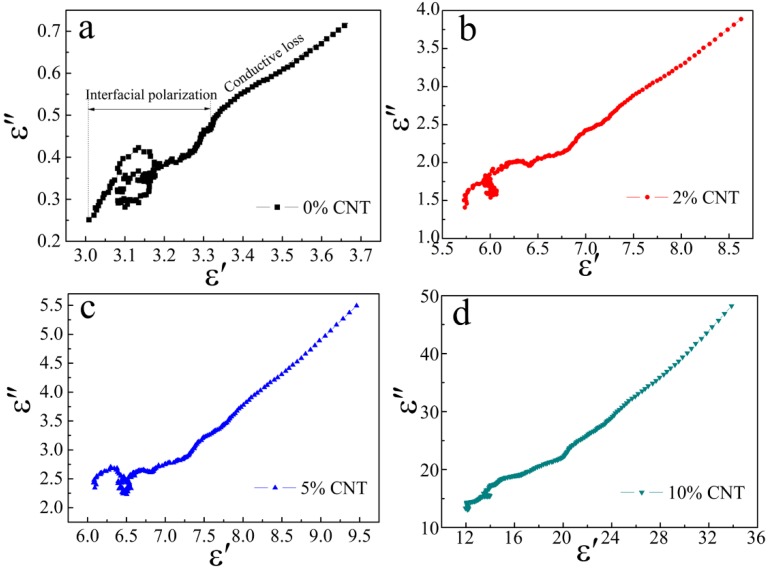
Plots of *ε′−ε″* for the composites. (**a**) 0% CNT; (**b**) 2% CNT; (**c**) 5% CNT; and (**d**) 10% CNT.

It can be deducted from the formula that the plot of *ε′* versus *ε″* would be a semicircle called the Cole-Cole semicircle. Each single semicircle denotes a Debye polarization relaxation course [34]. Figure 8 denotes the *ε′*–*ε″* plots of ZnO/Ni_3_ZnC_0.7_/x% CNT (x = 0, 2, 5, 10) composites in 2–18 GHz. All curves display three overlapped Cole-Cole semicircles, which represent three primary Debye relaxation processes in the composites. As indicated by Raman and XPS results, the carbon component contains abundant defects and some oxygen-containing functional groups, which can be considered as dipoles for converting the EM energy to the thermal energy due to the orientation relaxation process motivated by an alternating EM field. In addition, free charges that accumulate at the hetero-interfaces of composites will produce polarizations, which have an important effect on the electrical and dielectric properties. Under an alternating EM field, the accumulated charges produce Debye relaxation processes which attenuate the incident EM wave as well. The increase amount of CNT results in more CNT-carbon interfaces and therefore enhances interfacial polarization relaxations. In addition to the high electrical conductivity of CNT and carbon, conductive loss can be another significant factor for EM wave attenuation. It is shown in Figure 8 that the diameter of Cole–Cole semicircle decreases and the end part becomes longer with the increase of CNT content. The cole plots can be easily divided into two segments. These results indicate the contribution of conductive loss to the overall dielectric loss gradually increase with the improved graphitization degree of carbon and the increment of CNT. In addition, the multi-layer urchin-like structure of the composites might facilitate the scattering of incident waves, which is favorite for the increase of the interfacial polarization.

**Figure 9:**
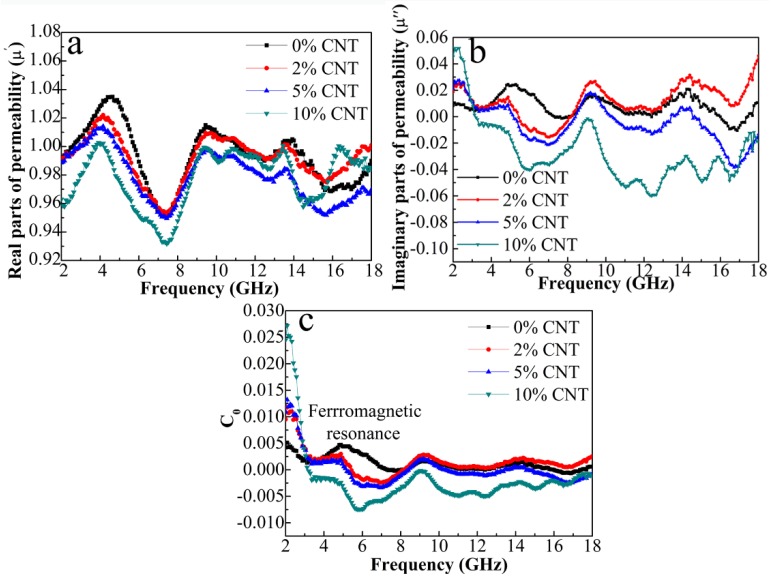
Frequency reliance of (**a**) *μ′*; (**b**) *μ″*; and (**c**) *C*_0_ for the composites.

**Figure 10:**
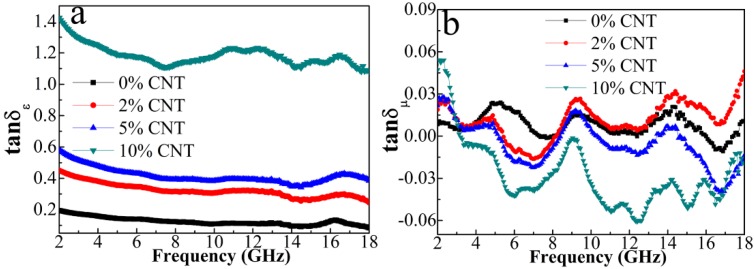
Frequency dependences of tan*δ_ε_* (**a**) and tan*δ_μ_* (**b**) of the four composites.

Meanwhile, we cannot neglect magnetic loss. Figure 9a shows the dependences of *μ*′ and *μ″* on the variable frequency. *μ′* and *μ″* values of the composites decrease as the CNT content increases, which is consistent with the trend of saturated magnetization in Figure 6. The *μ* value is related to the *M_s_* and magneto-crystalline anisotropy based on the Globus equation $\mu \propto {\left(M_{s}{}^{2}D/K_{1}\right)}^{1/2}$, where *D* is the grain size and *K*_1_ is the magneto-crystalline anisotropy constant. In this case, larger values of *M_s_* and grain size result in a larger permeability. It is known that the magnetic loss in gigahertz frequency mainly originates from eddy current loss, natural ferromagnetic resonance and exchange resonance. The resonant peaks of *μ″* curves in the low-frequency and the high-frequency region are derived from the natural ferromagnetic resonance and the exchange resonance, respectively. Moreover, the plot of *C*_0_ (*C*_0_ = *μ″*(*μ′*)^−2^*f*^−1^) versus frequency (shown in Figure 9c) is analyzed for reflecting the effect of eddy current on the magnetic loss. If the *C*_0_ value keeps constant with the frequency, it can be verified that the magnetic loss comes from the eddy-current loss [33]. Nevertheless, it is seen that *C*_0_ curves of all the composites strongly fluctuate over the entire frequency range. Therefore, the exchange resonance and natural ferromagnetic resonance rather than eddy current are primary factors for the magnetic loss. To further evaluate the dielectric loss and magnetic loss of *C*_0_/*C* composites, the loss tangent, i.e., tan*δ_ε_* = *ε″*/*ε′* and tan*δ_μ_* = *μ″*/*μ′*are calculated as shown in Figure 10. The tan*δ_μ_* values are almost below 0.06 while the tan*δ_ε_*varies in 0.93−1.04. The much larger tan*δ*_ε_ values demonstrate the dominant role of dielectric loss in EM wave attenuation in the whole frequency range.

**Figure 11:**
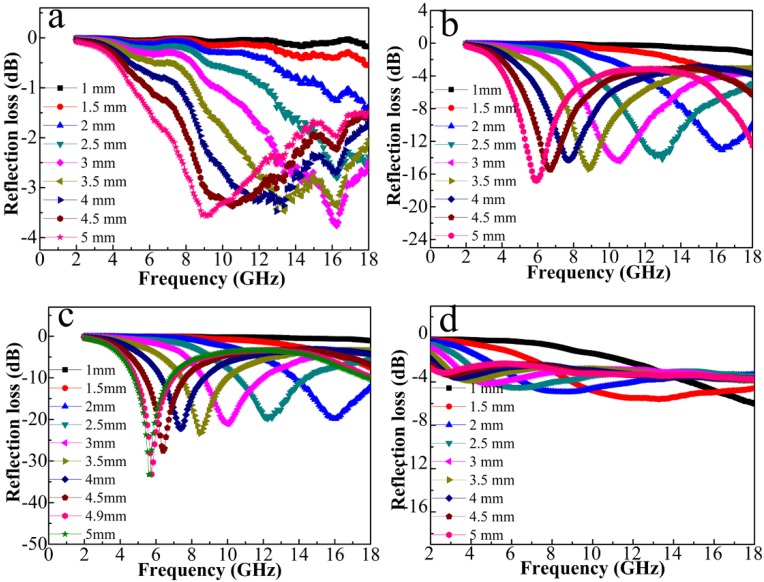
Frequency dependences of reflection losses of the composites: (**a**) 0% CNT; (**b**) 2% CNT; (**c**) 5% CNT; and (**d**) 10% CNT.

The absorbing property of the microwave in the samples can be assessed by the value of reflection loss, which can be calculated by using the measured complex permittivity and permeability according to the transmission line theory [35,36]

(2) $$Z_{\mathrm{in}}=Z_{0}\sqrt{\frac{\mu_{r}}{\varepsilon_{r}}}\tanh\left(j\frac{2\pi fd}{c}\sqrt{\mu_{r}\varepsilon_{r}}\right)$$

(3) $$\mathrm{RL}=20\;\log|\frac{Z_{\mathrm{in}}{-Z}_{0}}{Z_{\mathrm{in}}{+Z}_{0}}|$$

where *Z*_0_ is the characteristic impedance of free space, *Z_in_* is the input impedance of the absorber, and *c* is the light velocity. The microwave absorbing properties of the composites with different layer thicknesses are compared seen in Figure 11, from which we can observe the obvious impact of CNT amount on the composites. Among the four samples, 5% CNT composite exhibits the best microwave absorbing capability. When the layer thickness is above 2 mm, its reflection loss (RL) value exceeds −20 dB, which corresponds to 99% attenuation of incident EM waves. The RL value of 5% CNT composite is further optimized to a value of −33.2 dB at a layer thickness of 4.9 mm. Compared with previous multi-phase paramagnetic NPs/porous carbon absorber that derived from pyrolysis of MOF, 5% CNT composite shows the lowest filler loading and the lightest filling (shown in Table 1), which is highly desirable for the new-type microwave absorber.

**Figure 12:**
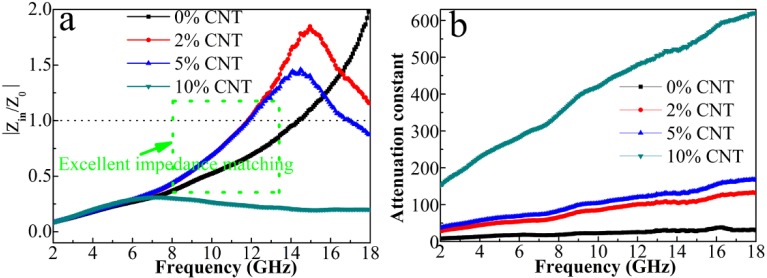
Frequency dependency versus frequency of (**a**) relative input impedance $\left|Z_{\mathrm{in}}{/Z}_{0}\right|$ and (**b**) attenuation constant α of the four samples.

**Figure 13:**
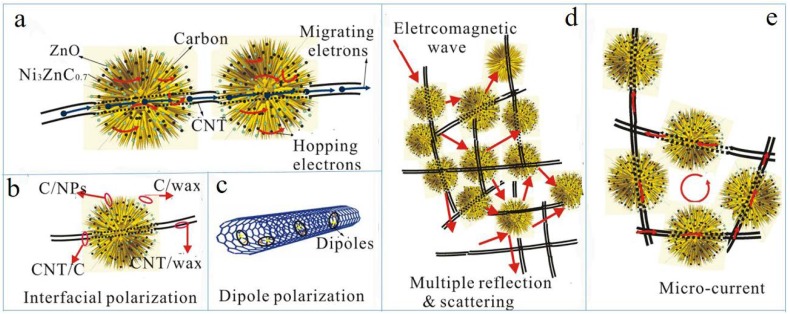
The pattern of electromagnetic wave attenuation models of the composites (**a**) hopping electrons (**b**) interfacial polarization (**c**) dipole polarization (**d**) multiple reflection and scattering (**e**) micro-current in the network.

To further explore the microwave absorption mechanism of the composite, two crucial factors including impedance matching (*Z*_in_/*Z*_0_) and attenuation constant ($\alpha =\frac{\sqrt{2}\pi f}{c}\times \sqrt{\left(\mu^{\prime \prime }\varepsilon^{\prime \prime }-\mu^{\prime }\varepsilon^{\prime }\right)+\sqrt{{\left(\mu^{\prime \prime }\varepsilon^{\prime \prime }-\mu^{\prime }\varepsilon^{\prime }\right)}^{2}+{\left(\mu^{\prime }\varepsilon^{\prime \prime }+\mu^{\prime \prime }\varepsilon^{\prime }\right)}^{2}}}$) are calculated as shown in Figure 12. To achieve outstanding microwave absorption performance, a better impedance matching degree and larger attenuation constant should be satisfied simultaneously [37,38,39,40,41,42,43]. The |*Z*_in_/*Z*_0_| value equal to 1.0 implies the zero-reflection of the incident EM wave at the front surface of absorber, which is required for the ideal impedance matching. As seen in Figure 12a, the |*Z*_in_/*Z*_0_| curve of 5% CNT composite is close to 1.0 in a wider frequency range as compared with other samples, which indicates the best impedance matching state among all the samples. Therefore, the incident EM wave will enter the 5% CNT absorber to a large extent, ensuring the dissipation of EM wave by the strong dielectric loss and magnetic loss inside the absorber as summarized in Figure 13. The improved impedance matching of 5% CNT composite derives from the precise modulation of permittivity and permeability by tuning the content of CNT and the particle size as well as the dispersity of multi-phase NPs. In addition, the high aspect ratio of CNT can lead to the formation of a conductive network in the paraffin matrix. It can be speculated that the contact conductivity mainly contributes to the *σ* of the network, which results from the electrons hopping process at the interfaces of the composites.

## 4. Conclusions

Novel microwave-absorbing composites of ZnO/Ni_3_ZnC_0.7_/CNT have been successfully synthesized by a facile thermal decomposition of Ni–Zn precursor MOF. The chemical state of each component and composition can be actually adjusted by changing the content of CNT. ZnO and Ni_3_ZnC_0.7_ NPs are relatively stable whereas physical and chemical properties of C element are tunable. The optimized 5% CNT composite exhibits better microwave absorption performance thanks to the well-designed composition and structure, as well as the synergistic effect between ZnO and Ni_3_ZnC_0.7_ NPs, C matrix, and CNT. Zn^2+^ and Ni^2+^ is transformed to ZnO and Ni_3_ZnC_0.7_, respectively, during the carbonization process, which leads to the graphitization and weight loss of carbon. When the filler loading of composite in the paraffin matrix is as low as 10 wt.%, the minimum RL value reaches −33.2 dB at the thickness of 4.9 mm. Interfacial polarization, conduction loss, dielectric loss, and ferromagnetic resonance are responsible for outstanding RL performance. This work offers an effective and facile method to prepare efficient and lightweight microwave absorbers derived from MOF precursors.

## Figures and Tables

**Table 1:** Microwave absorption properties of MOF-based absorbers reported in this work and recent literatures.

Filler	Matrix	Loading (wt.%)	Thickness (mm)	Minimum RL (dB)	Reference
ZnO/Fe/Fe_3_C/C	paraffin	60	1.5	−30.4	[10]
ZnO/SiC	paraffin	30	2.5	−31.3	[12]
ZnO@CNT/SiO_2_	paraffin	15	2.5	−20.7	[15]
Fe-Fe_3_C/C	paraffin	25	1.5	−17.9	[16]
ZnO/C@Co@C	paraffin	50	1.9	−28.8	[17]
ZnO/Co_3_ZnC/Co/C	paraffin	50	1.9	−32.4	[14]
ZnO/Fe	paraffin	60	1.5	−40	[13]
ZnO/Ni_3_ZnC_0.7_/5%CNT	paraffin	10	4.9	−33.2	this work

## Supplementary Materials

The following are available online at http://www.mdpi.com/2079-4991/8/8/600/s1, Figure S1: (a–d) elemental mapping images of 5% CNT composite; Figure S2: (a) XPS survey of the 5% CNT composite; (b) the crystal structure of Ni_3_ZnC_0.7_ particles; Figure S3: The picture of the atmosphere protection tube furnace in the experimental process; Figure S4: (a,b) TEM images of raw MWCNT(a) and acid treated CNT(b); Figure S5: Raman images of raw CNT and acid treated CNT; Figure S6: (a) The chemical structure of Ni_3_ZnC_0.7_ (b) The MOF structure of CCDC: 638866. Table S1: EDS results of the composites.
