# Supplementary material for: In-Situ Conversion of ZnO/Ni3ZnC0.7/CNT Composite from NiZn Bimetallic MOF Precursor with Enhanced Electromagnetic Property
Source: Nanomaterials (Basel). 2018 Aug 7;8(8):600. doi: 10.3390/nano8080600 (PMC6116268; doi:10.3390/nano8080600)
Supplement: Supplementary file 1 [file nanomaterials-08-00600-s001.pdf]

Supporting Information

In-situ conversion of $ZnO/Ni_3ZnC_{0.7}/CNT$ Nanocomposite from NiZn bimetallic MOF precursor with enhanced Electromagnetic Absorbing Property

[1] Shenzhen Key Laboratory of Laser Engineering, Guangdong Provincial Key Laboratory of Micro/Nano Optomechatronics Engineering, College of Optoelectronic Engineering, Shenzhen University, Shenzhen 518060, China; huanglina007@sina.com (L.H.); nkhsl3313@163.com (S.H.); yangziyu@pku.edu.cn (Z.Y.); ailunzhao@outlook.com (A.Z.); scruan@szu.edu.cn (S.R.)
[2] College of Mechatronics and Control Engineering, Shenzhen University, Shenzhen 518060, China; chxliu@szu.edu.cn
[3] State Key Laboratory of Silicon Materials, School of Materials Science and Engineering, Zhejiang University, Hangzhou 310027, China; lujianguo@zju.edu.cn
* Correspondence: yjzeng@szu.edu.cn; Tel.; +34-913941790; Fax: +34-913941786

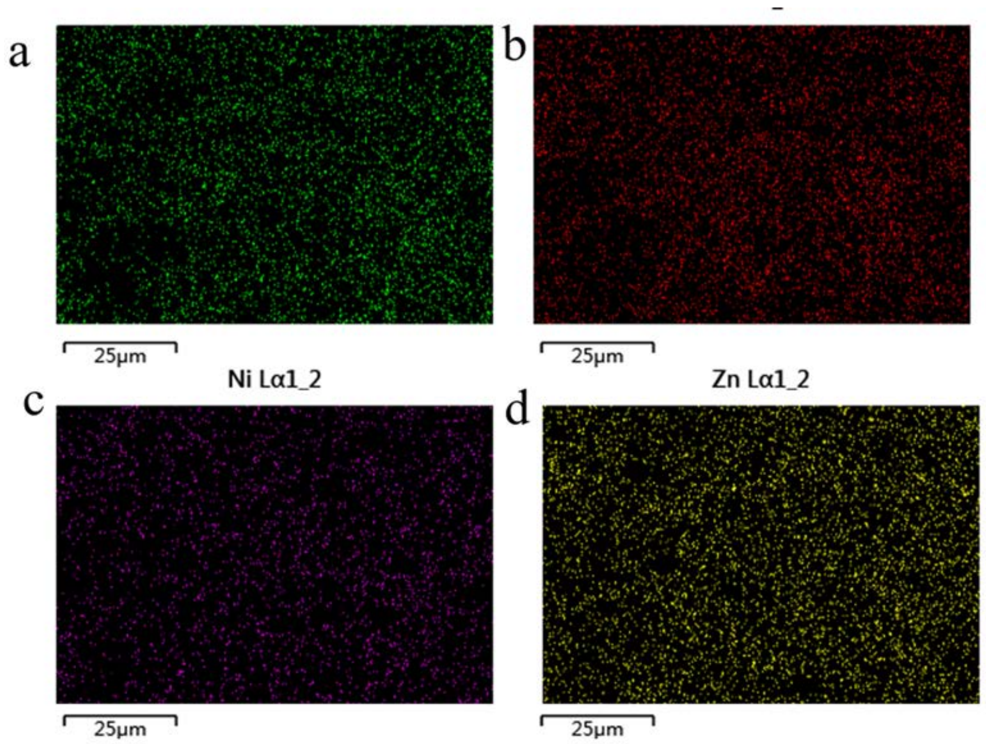

Figure S1. (**a–d**) elemental mapping images of 5% CNT nanocomposite.

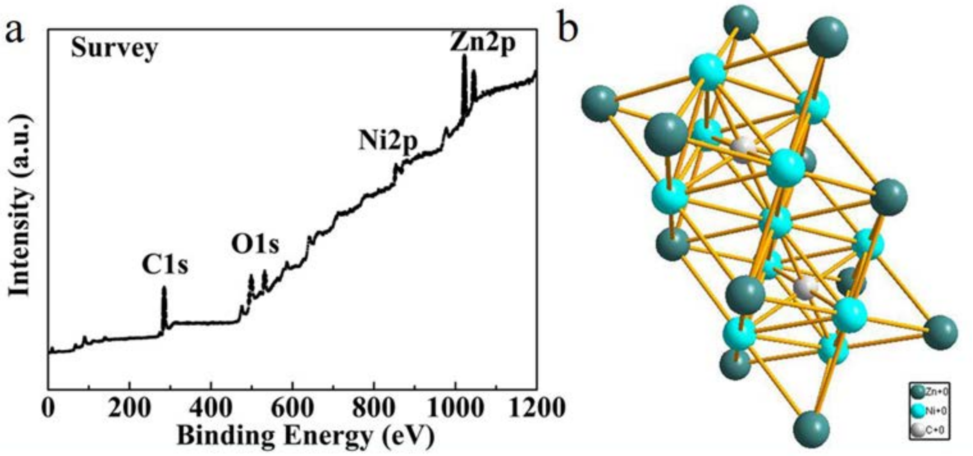

Figure S2. (**a**) XPS survey of the 5% CNT nanocomposite; (**b**) the crystal structure of $Ni_3ZnC_{0.7}$ particles.

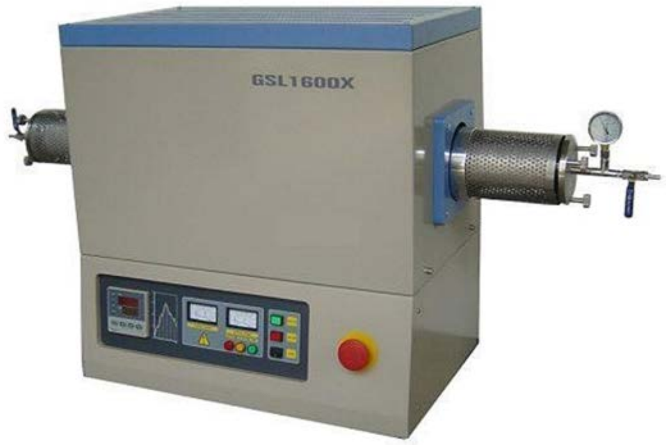

Figure S3. The picture of the atmosphere protection tube furnace in the experimental process.

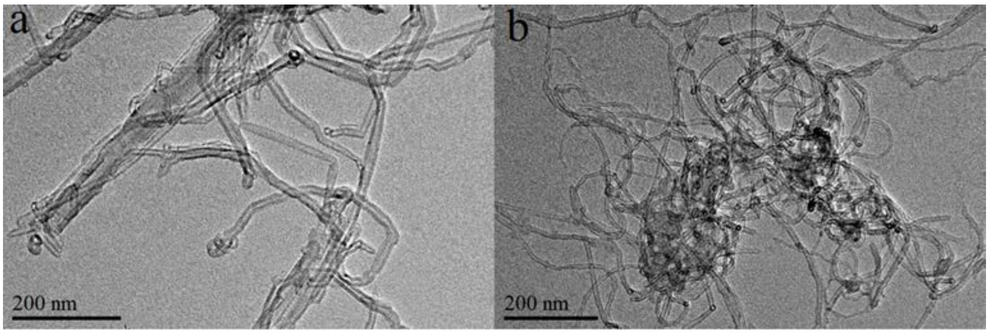

Figure S4. (**a**) TEM image of raw MWCNT; (**b**) TEM image of MWCNT after acid processing.

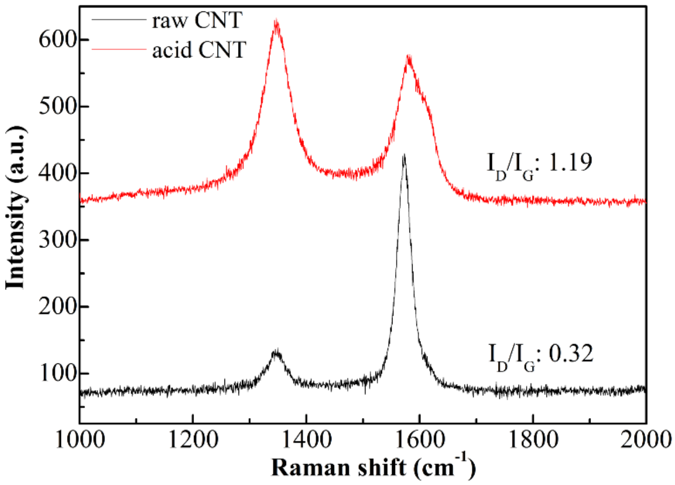

Figure S5. Raman spectra of raw MWCNT and acid treatment MWCNT.

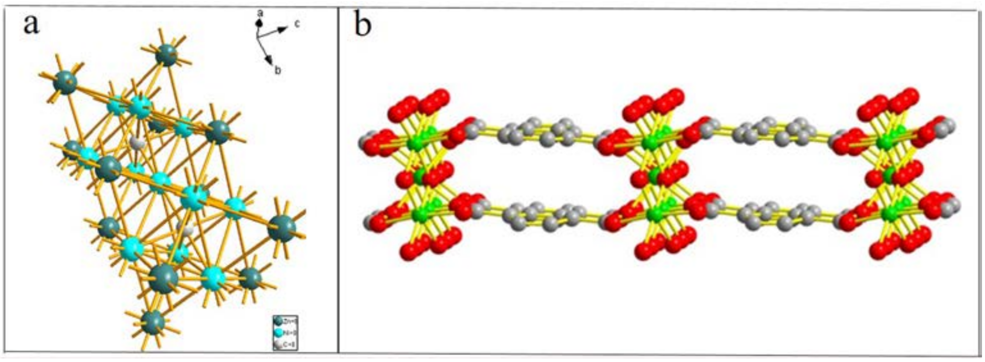

Figure S6. (**a**) The chemical structure of $Ni_3ZnC_{0.7}$; (**b**) The MOF structure of 638866.

Table S1. EDS results of the composites.

Sample	C (at.%)	O (at.%)	Ni (at.%)	Zn (at.%)
0% CNT	45.09	10.59	20.29	24.03
2% CNT	39.87	13.42	21.14	25.56
5% CNT	37.95	9.81	21.35	30.90
10% CNT	36.90	9.55	22.19	31.36
